# Fusion of Building Information and Range Imaging for Autonomous Location Estimation in Indoor Environments

**DOI:** 10.3390/s130202430

**Published:** 2013-02-14

**Authors:** Tobias K. Kohoutek, Rainer Mautz, Jan D. Wegner

**Affiliations:** Institute of Geodesy and Photogrammetry, ETH Zurich, Wolfgang-Pauli-Str. 15, 8093 Zurich, Switzerland; E-Mails: rainer.mautz@geod.baug.ethz.ch (R.M.); jan.wegner@geod.baug.ethz.ch (J.D.W.)

**Keywords:** indoor positioning, ToF cameras, range imaging, CityGML, point cloud library

## Abstract

We present a novel approach for autonomous location estimation and navigation in indoor environments using range images and prior scene knowledge from a GIS database (CityGML). What makes this task challenging is the arbitrary relative spatial relation between GIS and Time-of-Flight (ToF) range camera further complicated by a markerless configuration. We propose to estimate the camera's pose solely based on matching of GIS objects and their detected location in image sequences. We develop a coarse-to-fine matching strategy that is able to match point clouds without any initial parameters. Experiments with a state-of-the-art ToF point cloud show that our proposed method delivers an absolute camera position with decimeter accuracy, which is sufficient for many real-world applications (e.g., collision avoidance).

## 1. Introduction

Even though indoor positioning is a comparatively new research topic, the development of indoor positioning techniques has become a major research field. Three-dimensional (3D) geometries of objects or scenes need to be captured for a variety of applications, such as scene mapping, robot navigation, and video surveillance where physically deployed infrastructure should not be required during data acquisition to minimize the costs. Several hundred approaches have been made in the past few years, as no localization technology is able to cover the indoor space like Global Navigation Satellite Systems (GNSS) do for the open sky. In [1], Mautz summarizes the state-of-the-art in indoor positioning techniques. The majority of systems relies on active transmission of electromagnetic or sound waves and often approximate methods (proximity, scene analysis, *etc.*) are applied to obtain a rough estimate of the unknown location [2]. Beacon-based positioning techniques require knowledge of the geospatial location of their transmitters, which can be cumbersome to achieve. Laser scanners, measuring each point sequentially, triangulation methods (e.g., stereo-vision and photogrammetry), and interferometry are commonly used for optical based indoor positioning. Drawbacks of these techniques include time-consuming data acquisition due to the sequential scanning process of terrestrial laser scanners, challenging stereo image analysis for stereo camera systems or visual odometry [3], and limited depth range for interferometric methods [4]. Monocular vision systems based on smartphone camera images and their discovery in an image database [1,5] or floor plans [2,6] are difficult to interpret due to scale ambiguities. Additional information about landmarks (door frames, *etc.*) is needed and the expected accuracy of such technique is at meter level.

An alternative technique that is able to rapidly acquire large amounts of indoor depth data in a video-like fashion is range imaging (RIM). ToF range cameras measure depth information directly without need for stereo matching. Depth ranges of several tens of meters with centimeter to decimeter precision of state-of-the-art systems are largely sufficient for indoor applications.

We propose to estimate camera positions via matching of range image sequences to already available GIS data. Since relative orientations of objects and absolute position are known therein, we can use this information to pose our measuring device once newly acquired point clouds are accurately matched to the model. Such models have become widely available because various disciplines like Computer Aided Architectural Design (CAAD)/BIM, Computer Graphics, and Geographic Information Systems (GIS), which deal with 3D interior building models (e.g., IFC [3,7] or CityGML [4,8]). Note that such models do not only store 3D shape and position of objects, but represent their precise interior topography including semantics, too. For example, a cupboard in an office does not only appear as a cuboid but is explicitly tagged as a cupboard. Thus, single objects of interest for matching to newly acquired point clouds can rapidly be found in extensive datasets and can also be processed successfully.

Our method combines absolute and relative orientation to achieve decimeter accuracy of a mono-camera system (ToF range camera) while no dedicated markers nor any other locally deployed infrastructure (e.g., Wi-Fi hot spots) inside the building is required [9]. Moreover, no additional devices like inertial measurement units (IMU) or odometers are used. Matching and estimation of the camera position solely relies on range measurements and an a priori given building model. Note that a value adding service of our approach is the generation of a 3D building model from the observed point cloud.

In the following we first review works related to ours. After a conceptual overview of our approach, we provide a detailed methodological step-by-step explanation of the proposed point cloud matching and camera positioning procedure. Thereafter, experiments with a challenging dataset are described and discussed. Finally, we give conclusions and an outlook.

## 2. Related Work

In [10], Arman and Aggarwal proposed a definition for the exact estimation of location and orientation of an object, namely its pose, as the object recognition problem. Some prior knowledge about the object (e.g., shape, color, *etc.*) is relevant and can be contained in an object model. This model represents the object adequately if it is unique, not sensitive, unambiguous and convenient to use. Bosche and Haas [11] presented a model-based approach, which automatically registers 3D CAD models with laser scanner data. Therefore, the proprietary data format from CAD is converted into the Stereo-Lithography (STL) format and is then referenced in the laser scanner's spherical frame. The conversion to STL reduces computational complexity. Furthermore, the vertices of STL triangles are expressed with spherical coordinates.

In [12], Prusak *et al.* mounted a ToF range camera and a Fisheye camera to a mobile robot. The pose is estimated using a model-tracking/structure from motion (SfM) algorithm. In a first step range and fisheye images are mapped to a 3D-panorama to generate 2D-3D-correspondences therein. Further on, the 3D-points and estimated poses are used as input data for mapping the building based on a SLAM algorithm. Fuchs and May [13] reconstruct a cube's surface with an Iterative Closest Point (ICP) algorithm merging ToF range camera point clouds. The pose of the cameras was known a priori in a global coordinate system with a precision of 1 mm in translation and 0.1° in rotation. After depth and photogrammetric calibration of the cameras the cube was reconstructed during an ICP algorithm with an accuracy of approximately 3 mm in translation and 3° in rotation. In [14], Sheh *et al.* merged a generated point cloud with color and thermal image data. The ICP algorithm was used to generate textured and accurate maps of unstructured indoor environments. Therefore the data acquisition involved rotation by a pan-tilt unit, which took ten range images at intervals of 36°, stopping at each location long enough to avoid motion blur. However, a human operator, who identified landmarks, assisted the mapping procedure. Due to the drawback of ICP to often converge to an incorrect local minimum if the input point clouds are not already nearly aligned, May *et al.*[15] investigated modifications in a SLAM algorithm. They provide a performance benchmark comparing Kanade-Lucas-Tomasi feature tracker (KLT) and Scale-invariant feature transform (SIFT) algorithm to a depth-image based ICP algorithm and the hybrid Efficient Second Order Minimization (ESM) technique.

In [16] we have shown how to construct the object database in CityGML. CityGML is a standardized information model which considers the objects' geometry as well as their semantics, topology, and appearance [17]. In particular for the purpose of indoor modeling, the semantic model provides an object class ‘Room’ that contains attributes to classify rooms and their function, for example, as a living room or office. Objects' (e.g., installations, furniture) geometric relation/constellation and label (name) identify a specific room or at least minimize the possible number of rooms in a building in which the camera is located.

Based on this background we will present in this work how the transformation from acquired point clouds to an object model is realized. The main challenge of our approach is, that we face the problem of datasets with total different amounts of points. Furthermore, a 3D model needs to be matched to a 2.5D scan. In the present system only geometric information is used. The advantage of CityGML, its semantic data, can be used in future for room identification.

## 3. Autonomous Indoor Positioning

**Figure 1.**
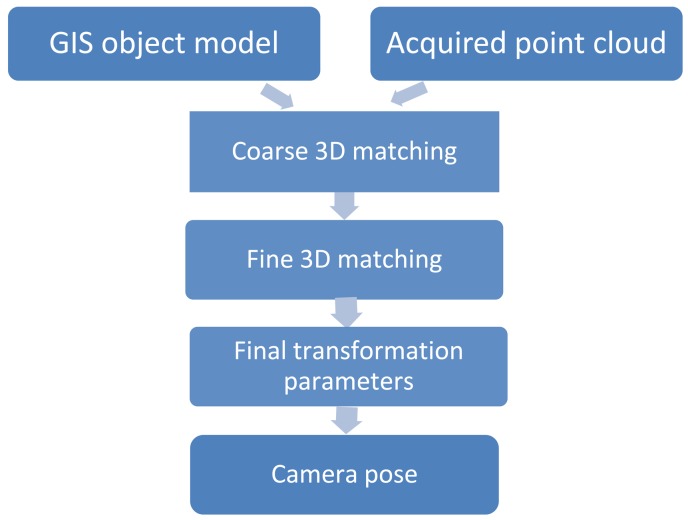
Overall concept of the presented approach.

The basic concept is to position a ToF range camera indoors via matching of range image sequences to GIS building models (Figure 1). A coarse-to-fine matching procedure consisting of three steps is developed and will be explained in the following. Range point clouds are transformed to the GIS model, where all relative and absolute point positions are known a priori, via a 3D transformation within an iterative matching framework. Once matching is accomplished the camera pose is estimated.

The database needs to be given a priori by a Building Information Model (BIM). Such models are nowadays established during the construction phase of a building and measured by other sensors, e.g., laser scanners. Recall that we neither assume any particular markers nor other locally deployed infrastructure inside the building. Although this assumption makes our method applicable to a wide range of objects and tasks, it makes sensor orientation and positioning challenging. Furthermore, the ToF range camera is used as a self-contained sensor where no additional information from other sensors as IMUs or odometers is used. Positioning solely relies on the range camera and a given building model.

### 3.1. Matching

A matching procedure for different spatial datasets being somehow located apart from each other always consists of two main parts: First, a suitable transformation that is capable of mapping the input to the reference and second, a metric that measures the fitting quality between both datasets after each transformation. Such metric can also be interpreted as a score function where an optimal score is aimed at. The exterior camera orientation is determined by a Cartesian 3D coordinate transformation with three shift and three rotational parameters. Transformation parameters are improved iteratively, for example using gradient descent methods, and after each run the metric measures the quality of fit. The goal is to find those transformation parameters that lead to an optimal fit between the datasets with respect to the metric. In practice, this sequence of transformation and metric evaluation is repeated iteratively until convergence of the metric value.

It should be noted that coordinates of the building model are usually given in an absolute national coordinate system (e.g., LV95 in Switzerland [18]) whereas camera range measurements are recorded in a local camera-specific reference system. It goes without saying that we initially have to convert both datasets, the acquired 3D object point clouds from the ToF range camera {*m_i_*} and the a priori known object models {*d_i_*}, *i* = 1…*N*, to the same reference system otherwise the following matching algorithms would not be applicable due to huge global coordinate offsets.

In a first step the absolute coordinates of the GIS object model are reduced such that the camera is located inside the building of interest. Therefore, the integer part of the first point of the object model is subtracted from all other model points thus accounting for large offsets. This translation $\left({\text{T}}_{i}^{G}\right)$ provides an initial guess for the translation vector *T_i_*. The entire following matching process then has the goal of estimating the camera's pose with respect to the GIS object model, *i.e.*, we match the ToF point clouds to the GIS object model. Once the global offset has been accounted for, we assume that input point cloud and target point cloud may have arbitrary orientation and position, both, relative to each other and in absolute coordinates. Literally speaking, we suppose that it is already known in which particular building the camera is located, but we do not know where exactly, e.g., in what room, on which floor *etc.* The transformation is calculated with:

(1) $${\text{d}}_{\text{i}}={\text{R}}_{i}{\text{m}}_{\text{i}}+{\text{T}}_{i}+{\text{V}}_{\text{i}}$$

where *R_i_* ∈ ℝ^3^ is a standard 3 × 3 rotation matrix, T_i_ is a 3D translation vector and V_i_ a noise vector [19]. Due to the fact that acquired point clouds and object models are metrical, a scaling operator is not needed. ToF cameras are able to measure the absolute distance [16]. Suitable reference points for the transformation (with six degrees of freedom) are the corners and walls of the room, vertices of doors, windows and other fixed installations or objects (e.g., furniture).

Generally, it is essential to keep prior assumptions as relaxed as possible because they could potentially limit applicability in practice. In a real-world scenario any kind of orientation and positioning from one point cloud with respect to the other one inside a building is possible and our method has to cope with this situation. Therefore, we use a coarse-to-fine matching procedure. A first coarse matching is done without need for precise initial values.

After the initial translation ${\text{T}}_{i}^{G}$ which basically shifts the ToF camera into the building of interest, the remaining displacement needs to be found. Most state-of-the-art algorithms establish correspondences between primitives of both datasets. A common solution for the registration problem is the Iterative Closest Point (ICP) algorithm or one of its variants [20]. ICP iteratively refines the relative pose of two pre-aligned point clouds by minimizing the sum of squared distances of corresponding points. Corresponding point pairs are identified via Euclidean distances of neighboring points in both scans. However, if point clouds are not pre-aligned with a certain precision, ICP tends to converge in a local minimum because nearest neighbor points do not correspond to the same points in the second point cloud if datasets are located far apart. In our case there is no pre-alignment of both point clouds because of the absence of any precise initial transformation parameters. Therefore, we cannot use ICP for matching.

A point cloud matching method without need for relatively precise initial transformation parameters is proposed in [21]. The two main advantages of the so-called Normal-Distributions Transform (NDT) are neither need for pre-alignment nor establishment of point correspondences between datasets for their registration. While ICP does a point-to-point matching, NDT is based on finding linear combinations of normal distributions. Normal distributions represent a piecewise smoothing of the scan and standard numerical optimization methods can be used for registration. Furthermore, computation time is increased because there is no need for nearest-neighbor search, which is a computational bottleneck of ICP. In [22], Magnusson extends the NDT registration to 3D and introduces some refinements. Such extended NDT algorithm uses a voxel data structure to represent local surfaces of objects compactly and carries out a More-Thuente line search [23]. The voxel grid data structure does not use individual points, but instead measures distribution statistics contained in each of its voxel cells to model point clouds as a set of multivariate Gaussian distributions. As a consequence, point cloud filtering as pre-processing step before registration is thus not necessary. NDT represents the probability of measuring a sample for each position. It is possible to adjust and optimize the probability of the existence of points at any position within the voxel. If the voxel size is chosen too small the registration will succeed only if point clouds are close to each other, similar to the pre-alignment for ICP. On the other hand it should not be chosen too big so that small objects can still be detected [24]. The lower cell size bound is given by the prerequisite to reliably compute a covariance matrix calling for at least five points per cell. A threshold (the epsilon parameter) is setup that defines the minimal change of the final transformation vector ( ${\text{T}}_{\text{i}}^{\text{NDT}}$ (x, y, z) and ${\text{R}}_{\text{i}}^{\text{NDT}}$ (roll, pitch and yaw)) [24]. The iterative alignment terminates when the incremental change reaches the threshold. The step length should shrink as it approaches the optimal solution. Larger distances can be covered by a smaller number of iterations using a larger maximum step length but at the risk of overshooting and ending up in an undesirable local minimum. We use the extended and refined NDT for coarsely registering ToF point cloud and GIS object model. It should be noted that the GIS object model originally only has points at plane intersections, which leads to several magnitudes less points than the ToF point cloud. Direct registration with such high point density difference is usually impossible and thus the GIS object model is augmented by randomly distributing points on each object plane.

**Figure 2.**
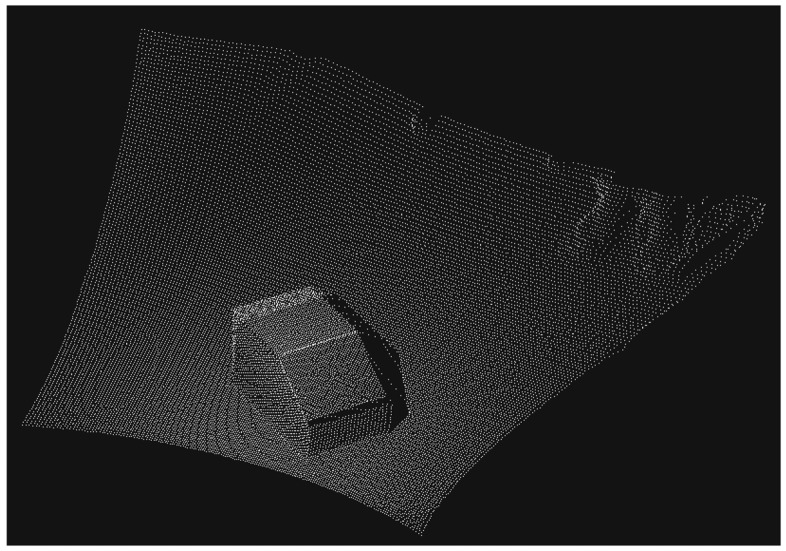
Acquired point cloud from ToF range camera.

Due to the fact that the point clouds have total different amounts of points, the NDT algorithm might not converge in its best solution. Therefore, NDT is used to achieve a coarse registration which is followed by fine registration with Correspondence Grouping (CG) [25]. The CG algorithm is capable to handle cases where the number of matched point correspondences is quite small compared to the total number of points like in our case. The object of interest is only a small part of the acquired ToF point cloud because the field of view of the sensor captures the entire environment like presented in Figure 2.

Due to the fact that the ToF point cloud contains not only points of the object of interest and the CG algorithm is not based on a voxel data structure like NDT the ToF point cloud needs to be filtered. A promising algorithm is given by plane model segmentation to delete for example the floor and walls. In [24], Okorn uses two clustering algorithms, one is based on a 3D Hough voting scheme [26], the other one is based on evaluating the consistency of the geometry [27]. We decided to focus on 3D Hough voting to detect free-form objects in range images because of the promising results of [26]. In the Hough voting approach random feature points and their local neighborhoods are extracted in GIS object model and ToF point cloud. By using a threshold, e.g., the Euclidean distance between points in a neighborhood, a set of correspondences can be determined, which is robust to wrong correspondences caused by noise or occlusions. Note that Hough voting in 3D space basically detects planes in the point clouds and results in matching of straight edges. The final transformation matrix includes values for the six degrees of freedom ( ${\text{T}}_{\text{i}}^{\text{CG}}$ (x, y, z) and ${\text{R}}_{\text{i}}^{\text{CG}}$ (roll, pitch and yaw)) as needed to transform the GIS object model to the local coordinate system of the ToF sensor. The final parameter set is passed to camera pose estimation.

### 3.2. Estimation of Camera Position

After the first translation of the object model coordinate system into the camera coordinate system the acquired point cloud will from now on be transformed into the object model. Once point clouds have been matched the camera pose can be estimated in a user specified interval (e.g., every 80 frames) by adding the translation vector ${\text{T}}_{i}^{G}$. The final transformation is calculated with:

(2) $${\left(\begin{array}{c} X\\ Y\\ Z \end{array}\right)}^{\text{Trans}}=\left(\begin{array}{c} T_{x}\\ T_{y}\\ T_{z} \end{array}\right)+\left(\begin{array}{ccc} \;1 & \;R_{z} & -R_{y}\\ -R_{z} & \;1 & \;R_{x}\\ \;\;R_{y} & -R_{x} & \;1 \end{array}\right){\left(\begin{array}{c} X\\ Y\\ Z \end{array}\right)}^{0}$$

with $T_{i}=\;{\text{T}}_{i}^{G}+T_{i}^{\text{NDT}}+T_{i}^{CG}$ and $R_{i}=R_{i}^{\text{NDT}}+R_{i}^{CG}$.

It has to be mentioned that the camera coordinate system of the MESA® ToF range camera is by construction not the same as usually used in the literature on perspective camera projection models. It lies in the center of the lens and X and Y are rotated around Z positively about *π*/2.

**Figure 3.**
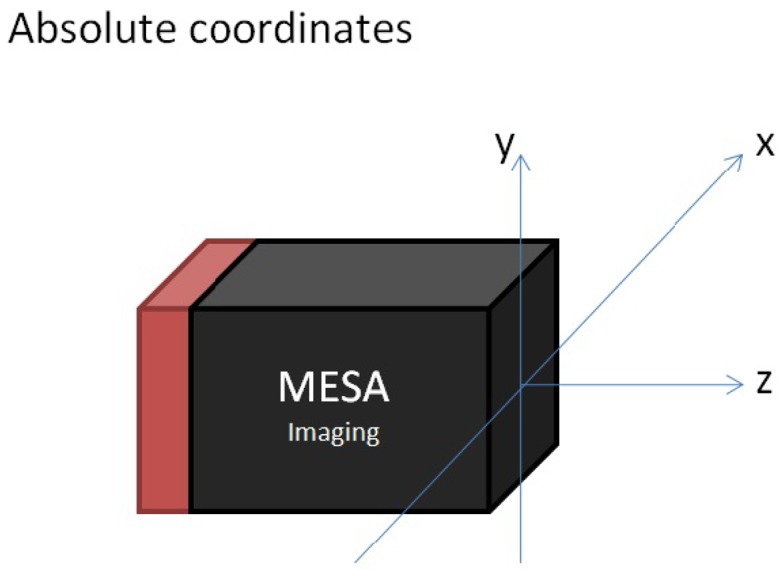
Origin (x,y,z) as delivered by the camera. Reproduced with permission from MESA Imaging AG, SR4000 User Manual; published by MESA Imaging AG, 2011 [28].

It is a “right-handed” camera coordinate system, with X-coordinate increasing horizontally to the left, Y-coordinate increasing vertically upwards and Z-coordinate increasing along the optical axis away from the camera. Figure 3 displays the origin of the coordinate system (0,0,0) which is located at the intersection of the optical axis with the front face of the camera.

## 4. Experiments

We implement all previously described algorithms in the framework of the open source Point Cloud Library (PCL) containing a wide range of state-of-the-art algorithms like filtering, registration, model fitting, *etc.*[29]. It offers well-elaborated algorithms in C++ and is also capable of handling 3D point clouds in real time.

We evaluate the proposed method on point clouds acquired with a MESA® ToF camera SwissRanger 4000 [30]. Acquired point clouds have an approximate 3D position accuracy of 1 cm for distances of up to 5 m and <1 dm accuracy for distances up to 15 m (in terms of a 1-σ standard deviation). For many indoor applications this level of accuracy is sufficient, e.g., collision avoidance. We chose a frame rate of 30 f/s to acquire a point cloud over all 25,344 pixels of the sensor.

**Figure 4.**
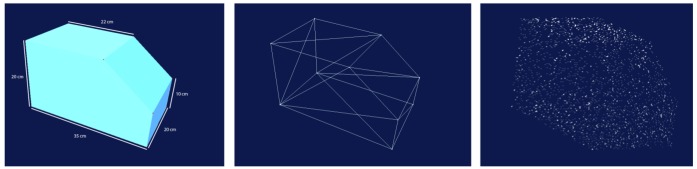
VRML model of the test object with seven surfaces and ten object points (**left**), as wire frame model (**middle**) and with added random points (**right**).

Our chosen test object is a block of wood that is uniquely identifiable in its orientation from all view directions. The object model was generated as a small VRML model in the Swiss coordinate system LV95. The VRML model is only represented by the ten edge points and the information which point is a member of which plane (Figure 4). However, the matching algorithm performs well with all other objects like installations and furniture. Nevertheless, the amount of points has to be increased to perform the matching algorithm successful because our approach calls for two point clouds with similar point densities as input. Therefore, up to 1,000 random points will be added to each plane of the object.

### 4.1. Sensor Specifications

**Figure 5.**
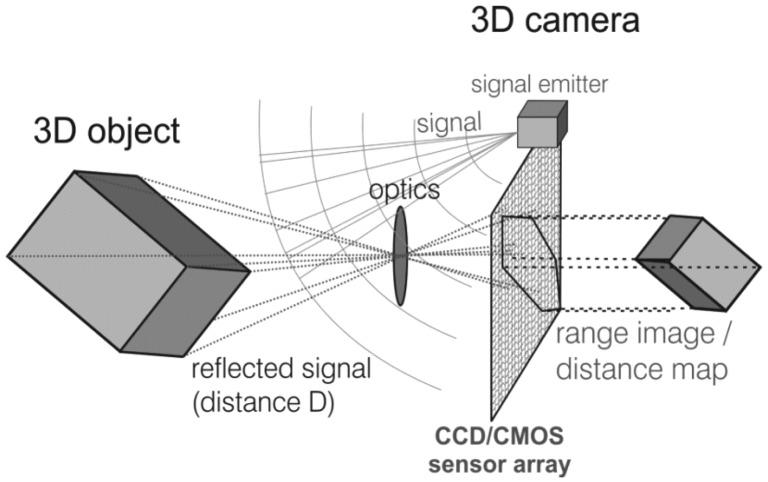
Time-of-flight principle. Reproduced with permission from T. Kahlmann and H. Ingensand, Proc. SPIE 6758; published by SPIE, 2007 [33].

The measuring principle of the MESA® ToF camera SwissRanger 4000, schematically shown in Figure 5, is based on the phase shift between light emitted from a light source and the reflected light received at a sensor using Complementary Metal Oxide Semiconductor technology (CMOS/CCD) [31]. The emitted light is pulsed at the modulation frequency *f*_mod_. The sensor samples the reflected light regularly and calculates the phase shift *φ* of the modulation with an autocorrelation function [32]. Since *φ* is proportional to the target range, it is possible to calculate an absolute target distance:

(3) $$D=\frac{c\;\varphi }{4\;\pi }$$

where *c* is the speed of light. In addition to the signal phase shift, the amplitude and the offset can be measured. Here, the amplitude indicates the strength of the modulated signal, which is an indication for the measurement accuracy. While the offset represents the local brightness of the scene, *i.e.*, a gray scale value similar to gray scale images.

The maximal non-ambiguity distance *D_max_* of 10 m is limited to half the modulation wavelength *λ_mod_*. Distances larger than *D_max_* are folded back to the non-ambiguity distance. Camera specifications of the device we use are listed in Table 1.

### 4.2. Camera Calibration

**Figure 6.**
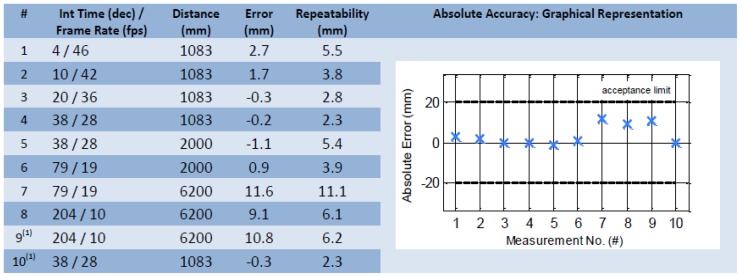
Final test results after calibration by manufacturer. All deviations are within the 20 mm tolerance. The absolute error represents the deviation between a reference distance and the distance measurements. Reproduced with permission from MESA Imaging AG, SR4000 Final Test Report; published by MESA Imaging AG, 2011 [34].

To increase the precision of the result the cameras interior orientation has to be determined previously. The SR4000 camera used had been calibrated by the manufacturer MESA® Imaging. Figure 6 shows the final test result from the manufacturer during an ambient temperature of 25°C (tests 9 and 10 performed at slightly higher housing temperature) without presence of background light.

The camera was given a warm up phase for at least one hour prior to data acquisition to ensure it reached internal temperature stability [35]. However, to reduce the signal to noise ratio (SNR) a mean point cloud was averaged over 100 measurements. The object was placed on the floor (dark brown colored carpet) and the ToF range camera was facing the object in a distance of 1.60 m from above (angle of incidence *ca*. 45°).

### 4.3. GIS Data Format

The a priori known GIS object models are stored as Virtual Reality Modeling Language (VRML) files with spatio-semantic information in CityGML [8] that supports any coordinate system and also provides the missing link between the indoor and outdoor space. VRML files represent the 3D geometry of objects in simple text files to keep the data storage small. The accuracy of the objects in CityGML is expected to be at centimeter level and should lead to position determination of the camera within centimeter accuracy. In CityGML geometries of variable components of a room can be modeled using so-called implicit geometries. Only a single instance of the object's shape is stored in the library in form of a VRML file even if multiple objects of the same shape are present (e.g., pieces of furniture). For each occurrence of such an object, only the local coordinates of an insertion point and a transformation matrix need to be stored in CityGML [16]. VRML was chosen instead of Extensible 3D (X3D) due to a smaller file size, which allows quick downloads of the object models via mobile Internet access. However, our approach is not restricted to VRML, objects could be modeled in X3D, too. GML database objects can be expressed in any coordinate system, in our case Swiss coordinates LV95 (e.g., X = 680,589.1000 m, Y = 251,368.1000 m, Z = 524.1000 m). The 3D Cartesian coordinate system of the acquired point cloud by the SR4000 is in metrical values too, but with a maximum value of around 10 m in indoor environments (e.g., X = 1.23 m, Y = 3.67 m, Z = 7.46 m).

### 4.4. Matching and Positioning Results

As we mentioned before, we chose a wooden block with plane surfaces as test object for our experiments. However, our proposed matching procedure works with any kind of object which is available in the database like cupboards, chairs, tables *etc.* Recall that our two-step matching procedure consists of an initial coarse registration applying the Normal-Distributions Transform (NDT) [21,22] followed by a fine registration via Correspondence Grouping (CG) [25].

In order to successfully register the point cloud acquired by the ToF camera with the GIS object model point cloud, the amount of points representing the model had to be increased to achieve roughly equal point densities in both datasets. The amount of additional model points was increased by randomly distributing between 300 and 1,000 points on each plane.

**Figure 7.**
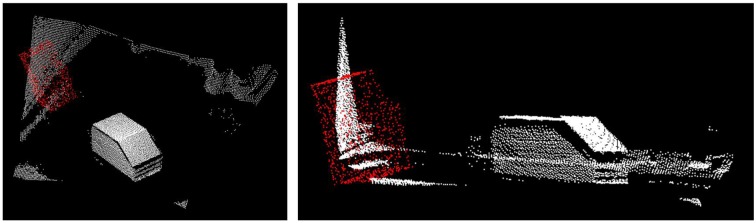
Acquired and model point cloud after ${\text{T}}_{\text{i}}^{\text{G}}$ translation (SR4000 point cloud without floor in white and model point cloud in red).

The modification of the scale dependent parameters of the NDT algorithm in our approach is based on the example in [24]. Figure 7 shows a possible example of the point clouds after translation ${\text{T}}_{\text{i}}^{\text{G}}$. In the following transformation steps the ToF point cloud is transformed to the object model.

We performed a grid search to find the optimal NDT parameter setting, which are shown in Table 2.

**Figure 8.**
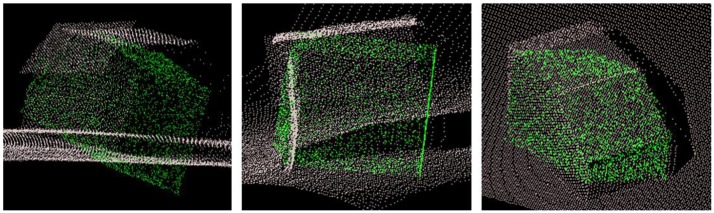
Solution of NDT algorithm (input data: object model point cloud in green and SR4000 point cloud in white).

Based on the chosen parameters, the algorithm took eight seconds to determine the location and calculate the transformation parameters ${\text{T}}_{i}^{\text{NDT}}$ and ${\text{R}}_{i}^{\text{NDT}}$. Figure 8 shows the acquired point cloud (white) transformed into the model point cloud (green). It remains a visible deviation in rotation. However, the NDT algorithm provides a good approximate solution that serves as input to a refinement with the CG algorithm.

The implemented CG algorithm is based on the tutorial of [25] that explains how 3D object recognition can be carried out using a PCL_Recognition module. In this work the algorithm based on 3D Hough voting scheme was used and the chosen parameters are presented in Table 3.

**Figure 9.**
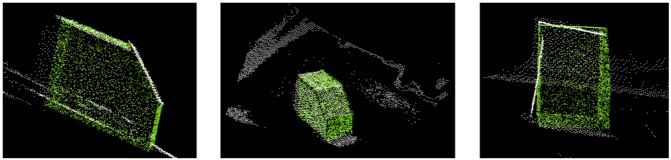
Solution of CG algorithm from different viewing angles; filtered SR4000 point cloud (white) and GIS object model point cloud (green).

Recall that our ToF point cloud covers much more than only the object itself. Thus we have to filter out non-relevant parts prior to fine registration. We adopt the plane model segmentation algorithm of [36], which is based on the number of points per surface with equal normal directions. Once surfaces have been segmented, all large ones with more points than a certain threshold alpha are assumed to belong to wall, floor or ceiling. Surfaces with fewer points are considered belonging to our object of interest located inside the room. A suitable threshold alpha is found empirically through multiple tests. In our case we achieve optimal results with alpha =1,600. All surfaces with more than 1,600 points are discarded. Figure 9 shows the result of the CG algorithm after filtering the input point cloud and using the solution from NDT algorithm as input. The CG algorithm converges in roughly ten seconds.

**Figure 10.**
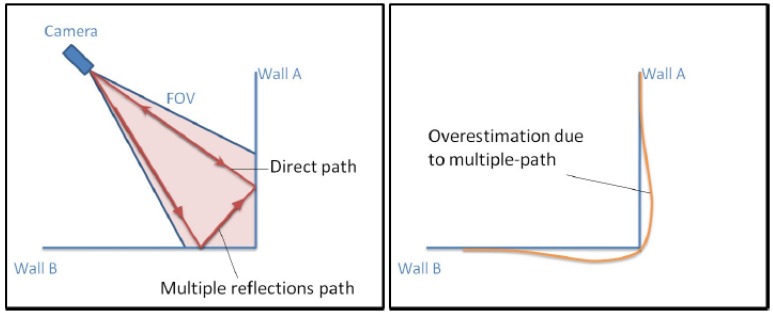
Multiple path reflections on a concave scene-in that case a corner between two walls. Reproduced with permission from MESA Imaging AG, SR4000 User Manual; published by MESA Imaging AG, 2011 [28].

The model point cloud fits significantly better to the SR4000 point cloud than the original output from the NDT algorithm does (Figure 7). Due to the fact, that there are no identical points in the model object and acquired point cloud a quality for point correspondences cannot be given. Depending on the input data, other, more unique information like cluster of points and/or empty spaces and features give a better estimation for the matching quality than points. However, like in NDT algorithm a small but visible deviation in rotation remains after the transformation ( ${\text{T}}_{\text{i}}^{\text{CG}}$ and ${\text{R}}_{\text{i}}^{\text{CG}}$). This can be explained through data acquisition and multipath reflections. A large portion of the infrared light is reflected first on the floor and then on the measured object into the camera. The region of the wall in Figure 10 will be seen further away than it is in reality and turns out in a concave structure (see orange line).

Maximum overestimation is in the region where multiple reflection paths are both, maximum in number and in intensity. This explains the shape of the measured values, as shown in orange on Figure 10 (right hand side). Due to the fact that the light travels the direct and the indirect path the apparent distance is then a weighted average of the paths, weighted by the signal strengths. This results in over-estimated distance measurements.

The Template Alignment algorithm (TA) presented in [37] was additionally tested to determine the object's position and orientation. TA uses a 3D template (the object model) as input and aligns it to the target cloud by applying the Sample Consensus Initial Alignment (SAC-IA) method to align source to target. The sample consensus method samples large numbers of correspondences and ranks them very quickly [38]. It maintains the geometric relations of the correspondences without testing all combinations. The maximum correspondence distance is specified as the squared distance with a value of 2 cm^2^. After calling a SAC-IA's accessory method the final transformation matrix ( ${\text{T}}_{\text{i}}^{\text{TA}}$ and ${\text{R}}_{\text{i}}^{\text{TA}}$) and fitness score are obtained. Fitness scores indicate the matching quality. It can readily be used as evaluation criterion where smaller values indicate better matching results [37].

The template alignment algorithm takes the longest computation time compared with the other two tested algorithms and did not work with our input data; the ToF point cloud was transformed to a completely wrong location. We conclude that this is a result of the large difference in the density of points between the two input point clouds causing the failure of the TA approach. In order to provide a better initial solution for the algorithm the input model point cloud was exchanged with the outputpoint cloud from the NDT algorithm, like it was done in the GC algorithm. Nevertheless, there was no improvement.

## 5. Conclusions

Efficient absolute positioning of a ToF range camera based on object acquisition in form of measured points in 3D Cartesian coordinates is possible. The absolute position of the camera can be calculated with decimeter accuracy based on the transformation parameters obtained in the presented coarse and fine registration with NDT and CG algorithm. The position of the ToF camera can be transformed into the reference coordinate system, *i.e.*, the coordinate system of the spatio-semantic 3D model. The possibility of using original VRML text format, which allows data compression for the purpose of quick download from the Internet and keeping the database small-sized.

However, ToF cameras still suffer from a set of error sources that hamper the goal of infrastructure-free indoor positioning. State-of-the-art range imaging sensors measure distances unambiguously between 0.5–10 m at an accuracy level of centimeters. Besides the influence of incidence angle [39] and scattering artifacts [4,40] the distance measurement errors are also a result of differing reflectivity of objects in the scene [41]. It can be seen in the input SR4000 point cloud, where vertical planes in reality are not vertical in the acquired point cloud. This is due to multipath reflections. Another influence is given by the reflective properties of the model object like material, color and gloss. Furthermore, we did no self-calibration of the camera.

Nevertheless, the algorithms provided in the point cloud library can deal with any kind of point clouds. The generated point clouds from ToF cameras are small in their data amount in comparison to laser scanner data and can therefore be processed in near real time. Due to fast processing of 3D data at high frame rates, ToF cameras are well suited for kinematic applications, such as 3D obstacle avoidance, gesture recognition [42] or generating indoor maps [14,15].

## 6. Outlook

The proposed location approach using only a range imaging sensor could be improved by self-calibration as well as considering additional sensors. Adding observations of an electronic compass and/or a tilt sensor would provide approximate values of some transformation parameters and therefore stabilize the search for the correct transformation set. Furthermore, if our approach will be used for an UVS equipped with a ToF camera additional problems like so-called mixed pixels or motion artifacts have to be solved by using filtering methods (e.g., [43,44]). Furthermore, the usage of spatial and semantic information of CityGML can be expended for pose estimation. The spatial relation of objects can help to estimate the pose more robustly, for example, if another object occludes one object but its surroundings can be detected. In such case the semantic information could support room identification, for example, if the amount of objects (e.g., tables, monitors, *etc.*) inside a room is determined and compared with the database. The current demonstrator implementation determines the camera pose, (position and rotation) offline on a consumer PC. The future goal is to run the location estimation on a mobile device with data link to the ToF range camera and to the object database.

## Figures and Tables

**Table 1.** SR4000 specifications (modified from [30]).

**Modulation frequency (MHz)**	**14.5–31**
Measurement range (m)	calibrated 0.8–8
Sensor pixels	176 × 148
Field of view (degree)	43.6 × 34.6
Scan resolution at 3 m (mm)	13.6
Footprint area at 3m (m^2^)	4.48
Camera weight (g)	470
Camera dimensions (mm)	65 × 65 × 68
Frame rate (f/s)	54
Illumination wavelength (nm)	850
Price (€)	∼5500

**Table 2.** Parameters of NDT used to produce Figure 8.

pcl∷NormalDistributionsTransform<pcl∷PointXYZ, pcl∷PointXYZ> ndt;	
ndt.setTransformationEpsilon (0.01);	// min. transform. difference for termination condition (m)
ndt.setStepSize (0.3);	// max. step size for More-Thuente line search (m)
ndt.setResolution (2.0);	// resolution of NDT grid structure (VoxelGridCovariance) (m^2^)
ndt.setMaximumIterations (100);	// max. number of registration iterations.

**Table 3.** Code example for the used CG algorithm.

// Compute Descriptor for keypoints
pcl∷SHOTColorEstimationOMP<PointType, NormalType, DescriptorType> descr_est;
descr_est.setRadiusSearch (0.1f); // unit: (m)
// Clustering
pcl∷Hough3DGrouping<PointType, PointType, RFType, RFType> clusterer;
clusterer.setHoughBinSize (0.07f);
clusterer.setHoughThreshold (16.5);
